# Association between TGFBR1*6A and osteosarcoma: A Chinese case-control study

**DOI:** 10.1186/1471-2407-10-169

**Published:** 2010-04-29

**Authors:** Yun-Sheng Hu, Yong Pan, Wen-Hai Li, Yong Zhang, Jun Li, Bao-An Ma

**Affiliations:** 1 Center of Orthopaedic Surgery, Orthopaedic Oncology Institute of PLA, Tangdu Hospital, Fourth Military Medical University, Xi'an, China; 2 Department of Plastic Surgery, Xijing Hospital, Fourth Military Medical University. Xi'an, China; 3 Department of Thoracic Surgery, Tangdu Hospital, Fourth Military Medical University, Xi'an, China

## Abstract

**Background:**

TGFBR1*6A is a common hypomorphic variant of transforming growth factor β receptor 1 (TGFBR1). TGFBR1*6A is associated with an increased cancer risk, but the association of this polymorphism with osteosarcoma remains unknown. We have measured the frequency of TGFBR1*6A variants in osteosarcoma cases and controls.

**Methods:**

Our case-control study is based on 168 osteosarcoma patients and 168 age- and gender-matched controls. Blood samples were obtained and the TGFBR1*6A variant determined by PCR amplification and DNA sequencing. The odds ratio (OR) and 95% confidence interval (95% CI) for the TGFBR1*6A polymorphism were calculated by unconditional logistic regression, adjusted for both age and gender. Three models - dominant, additive and recessive - were used to analyze the contribution of the TGFBR1*6A variant to osteosarcoma susceptibility.

**Results:**

Heterozygotic and homozygotic TGFBR1*6A variants represented 50.4% and 6.0% of the 168 cases, whereas the controls had 18. 5% and 1.3%, respectively. ORs for homozygosity and heterozygosity of the TGFBR1*6A allele were 4.6 [95% CI, 2.33-7.97] and 2.9 [95% CI, 1.59-5.34] in the additive model. There were significant increases in the TGFBR1*6A variants in osteosarcoma cases compared to control in all 3 models. Further analysis showed that TGFBR1*6A genotypes were not associated with gender, age, or tumor location. However, TGFBR1*6A was significantly associated with less metastasis.

**Conclusions:**

TGFBR1*6A, a dominant polymorphism of TGFBR1, is associated with increased susceptibility and metastasis spread of osteosarcoma.

## Background

Osteosarcoma is the primary malignancy of bone that has peak occurrences in adolescence and after 50 years of age [1,2]. Although there have been many studies on its genetics, biology, pathology and clinical aspects, the etiology of osteosarcoma is not well understood. Screening large series of children with osteosarcoma showed that ~4% carried a constitutional germline mutation in p53, suggesting a genetic predisposition of osteosarcoma [3].

TGF-β signaling plays an important role in the development of tumors because it is a potent inhibitor of cell proliferation in most cell types [4,5]. Tumors frequently lose responsiveness to TGF-β-mediated growth inhibition due to disruption in the TGF-β signaling pathway [6]. TGF-β regulates gene expression and enzymes activity in osteosarcoma, and modulates the effects of hormones on osteosarcoma, suggesting that TGF-β signaling is involved in the development of osteosarcoma [7-9]. TGF-β binds to its receptor, TGF-β receptor 2 (TGFBR2), which forms a heteomeric complex with TGF-β receptor 1 (TGFBR1). This leads to the transduction of the TGF-β signal from the cell surface to the cytoplasm [10,11]. Mutation of TGFBR1 may result in a high risk of certain cancers. For example, TGFBR1*6A is a common mutation of TGFBR1, and this variant is generated by deletion of 3 GCG triplets that coding for alanines within a 9 alanine (*9A) sequence at exon 1. TGFBR1*6A mutation can lead to the development of breast cancer, ovarian cancer, and renal cell carcinoma amongst others [12-15]. However, the relationship between TGFBR1*6A mutation and osteosarcoma occurrence remains unknown. There have been no reports about the prevalence of TGFBR1*6A mutation in osteosarcoma. Clarification of the relationship between TGFBR1*6A and osteosarcoma may indicate a role of TGF-β signaling in the etiology of osteosarcoma and provide clues that might help to guide treatment of this diseases. To clarify this relationship, we have analyzed TGFBR1 variation in clinical cases of osteosarcoma and normal controls. TGFBR1*6A positively correlated with the occurrence of osteosarcoma.

## Methods

### Study Subjects

Patients with osteosarcoma admitted to the Department of Orthopedics at Tangdu Hospital from January 2001 to March 2009 were selected to participate in the study, making a total of 168 patients. The mean age in the cases was 30 years (range 10-71 years). All the cases were diagnosed without a familial cancer syndrome. As controls, DNA from 168 blood donors of mixed gender collected at Tangdu Hospital, Xi'an, China, were used. The controls were disease-free. Informed consent was obtained from all the subjects, and the study was approved by the ethical committee at Tangdu Hospital.

### TGFBR1*6A genotyping

TGFBR1*6A genotyping invovled the protocol of Song et al [14]. PCR amplification of exon 1 of TGFBR1 was performed with the following primers: Forward 5'-GAG GCG AGG TTT GCT GGG GTG AGG-3' and Reverse 5'-CAT GTT TGA GAA AGA GCA GGA GCG-3'. The reactions were based in the protocol given by Invitrogen (Paisley, UK) in a total volume of 25 μl containing 50 ng DNA and 1.25 U Platinums Taq DNA polymerase. Fluorescently labeled PCR products were separated with an ABI 377 DNA Sequencer. Genotypes analysis was done with the GENESCANTM and GENOTYPERTM software. A product size of 247 bp represented the wild-type TGFBR1 (9A) allele, whereas a product size of 255 bp represented the TGFBR1*6A allele.

### Statistical analysis

Genotype distribution in osteosarcoma cases and controls was tested for the Hardy-Weinberg equilibrium, and the 2 variants were shown to be in equilibrium in both the patients and the controls. Differences in TGFBR1*6A variant carrier frequencies between cases and controls for the dominant and recessive models were assessed by the χ^2 ^test. The Armigate treand test was used to calculate *P *for trends in the additive model. Results are presented as odds ratios (ORs) with 95% test-based confidence intervals (CIs). All *P *values were 2-sided and *P *< 0.05 was considered significant.

## Results

### TGFBR1*6A genotypes and osteosarcoma susceptibility

The frequencies of 9A/6A and 6A/6A genotypes were significantly different between the cases and controls. In 168 cases, 9A/9A, 9A/6A and 6A/6A genotypes re[resented 107, 51 and 10, respectively. In 168 controls, the genotypes of 9A/9A, 9A/6A and 6A/6A were 134, 31 and 3, respectively. Heteozygotic and homozygotic TGFBR1*6A variants were 50.4% and 6.0% in total 168 cases, while they were 18.45% and 1.29% respectively in total 168 controls. When the data were analyzed with the dominant, additive and recessive models, there were significant increases in TGFBR1*6A variants in osteosarcoma cases compared to control in all of the (P < 0.01). The result are shown in Table 1.

**Table 1:** TGFBR1*6A genotypes in controls and patients with osteosarcoma

TGFBR1*6A genotypes	Cases (n = 168) number (%)	Controls (n = 168) number (%)	OR [95% CI]	*P *value
**Dominant model**				
9A/9A	107 (63.69)	134 (79.76)	1 (ref)	
9A/6A and 6A/6A	61 (36.31)	34 (20.24)	3.48 [1.92-6.17]	0.0011*
**Additive model**				
9A/9A	107 (63.69)	134 (79.76)	1 (ref)	
9A/6A	51 (30.36)	31 (18.45)	2.91 [1.59-5.34]	
6A/6A	10 (5.95)	3 (1.79)	4.59 [2.33-7.97]	0.0018**
**Recessive model**				
9A/9A and 9A/6A	158 (94.05)	165 (98.21)	1 (ref)	
6A/6A	10 (5.95)	3 (1.79)	4.03 [1.99-6.87]	0.0001*

* χ^2^-test; ** *p *for trend (Armitage's treand test)

### TGFBR1*6A genotypes and clinical parameters of osteosarcoma

No significant statistic differences were shown in the association of TGFBR1*6A genotypes with clinical parameters between males and females. Nor was any significant difference found between patirnts under 20 and those over 20. The location of osteosarcomas in long tubular bones and the axial skeleton was not significantly associated with a particular mutation of the TGFBR1*6A genotype. However, in cases with distant metastasis, the frequency of TGFBR1*6A genotypes was significantly decreased compared with those without distant metastasis (P < 0.01). The result are shown in Table 2.

**Table 2:** Associations of TGFBR1*6A genotypes with clinical parameters

Characteristics	Total Cases (n = 168)	TGFBR1*6A genotypes		*p *value
			
		9A/9A (%)	9A/6A + 6A/6A (%)	
***Gender***				
Male *	103	66 (64.08)	37 (35.92)	
Female	65	41 (63.08)	24 (36.92)	0.99
***Age***				
> 20 *	49	32 (65.31)	17 (34.69)	
≤ 20	119	75 (63.03)	44 (36.97)	0.86
***Location***				
long tubular bones *	137	86 (62.77)	51 (37.23)	
axial skeleton	31	21 (67.74)	10 (32.26)	0.13
***Distant metastasis***				
Without *	140	85 (60.71)	55 (39.29)	
With	28	22 (78.57)	6 (21.43)	0.001

* were used as the reference.

## Discussion

The 9A/6A and 6A/6A genotypes of TGFBR1 have a significantly higher frequency in osteosarcoma cases than in controls. TGFBR1*6A is a dominant susceptibility allele in the occurrence of osteosarcoma. The retrospective, non-randomized nature of most case-control studies limits the conclusion that can be reached from them. Because the subjects our study were from different regions of China for a long period from 2001 to 2009, the results have been more informative.

The association between TGFBR1*6A and tumorigenesis seems to be different in our case. Previous reports indicated that TGFBR1*6A increased susceptibility of tumorigenesis and development in renal cell carcinoma, ovarian cancer, and breast cancer [12-14,16], although others found no association of TGFBR1*6A with the occurrence of lung cancer, prostate cancer, colon cancer and bladder cancer [17-21]. Thus TGFBR1*6A genotypes mya be specifically related to only certain kinds of cancer, in a population- and tissue-related manner.

TGF-β signaling may not be the same in different tissues, which might explain the tissue-related manner of the association between TGFBR1*6A genotypes and cancer development. TGF-β signaling seems to be involved in tumorigenesis because it may behave aberrantly in its role as a potent inhibitor of cell proliferation in most epithelial and lymphoid cells [22]. Tumors frequently lose responsiveness to TGF-β-mediated growth inhibition due to disruption of its signaling pathway [23]. Loss of expression of TGF-β receptors may be as a prognostic factor in patients with renal cell carcinoma [24]. Receptor interactions at the level of the plasma membrane have been implicated in the regulation of TGF-β signaling pathway. In a mice model, constitutively reduced TGFBR1-mediated TGF-β signaling significantly enhances colorectal cancer development and more rapid tumor cell proliferation [25]. Tissue-specific expression of TGF-β receptors could explain the varying effects of TGF-β in different tissues. For example, targeted deletion of TGF-β receptors in mouse mammary epithelium results in excessive lobular-alveolar cell proliferation [26], but in contrast, no developmental changes were apparent upon deletion of TGF-β receptors in the epithelia of the oral cavity, esophagus, forestomach, pancreas and intestine of mice [27-29]. The unresponsiveness of some tissues to TGF-β signaling may explain their insensitivity to TGFBR1*6A variant, and vice versa.

Our study firstly demonstrates the significant association between TGFBR1*6A and osteosarcoma, which may be due to the important role of TGF-β signaling in the control of osteosarcoma. Some data indicates that TGF-β can be an important factor in the occurrence of osteosarcoma. TGF-β receptor is expressed in osteosarcoma [30]. TGF-β increases alkaline phosphatase (AP) activity in the rat osteoblastic cell line, ROS 17/2.8, and may promote osteoblastic differentiation in rat osteosarcoma cells [31]. We found that TGFBR1*6A genotypes are significant associated with osteosarcoma, which lends support to the fact that TGF-β signaling plays a vital role in the occurrence of osteosarcoma. The TGFBR1*6A allele encodes a type I receptor of TGF-β with reduced growth-inhibitory signaling activity [32]. The genetic variant of TGFBR1*6A could represent a functional modification in the TGF-β signaling pathway in osteosarcoma. Although this may not be necessary in determining the occurrence of osteosarcoma, TGFBR1*6A variants appear to increase *susceptibility *to osteosarcoma.

Osteosarcoma has a high incidence in young children and is often located in long tubular bones [33]. Therefore, we analyzed the association of TGFBR1*6A with some clinical parameters including gender, age, location and distant metastasis. Groups are usually divided at the age of 20, We found no significant association between the two groups above and below this age, and therefore TGFBR1*6A variants are not associated with age. In addition, TGFBR1*6A variants is not associated with the location of osteosarcoma, i.e. when frequency in long tubular bones and axial skeleton were compared. Therefore, TGFBR1*6A variants increased the susceptibility of osteosarcoma universally but not specifically to some cases with different gender, age and location of the tumor. This indicates that TGFBR1*6A makes for some functional modification of TGF-β signaling which stimulates the occurrence of osteosarcoma without regard to gender, age and location.

Analysis of distant metastasis, however, showed that TGFBR1*6A is significantly related with metastasis in osteosarcoma. The decreased risk of distant metastasis of osteosarcoma in TGFBR1*6A variants suggests that TGF-β signaling is involved in the metastasis of osteosarcoma. Tumor metastasis is a complex process and many factors are involved in this process, TGF-β is only one of these factors. For example, TGF-β promotes tumor metastasis in cancer [34-36]. Although the functional variants of TGFBR1*6A increases susceptibility to osteosarcoma, it decreases the probability of metastasis. TGFBR1*6A has been shown to enhance the migration and invasion of MCF-7 breast cancer cells [37]. Our finding of decreased metastasis in osteosarcoma cases with TGFBR1*6A contrasts with the result with in breast cancer cells. Since tumor cells may exhibit mutations, making their genotypes different from normal tissues, we examined the possibility of loss of homozygosity of TGFBR1 in osteosarcoma tissues in 10 cases with distant metastasis. TGFBR1*6A genotypes were the same as observed with blood samples (data not shown), with no loss of homozygosity of TGFBR1 in the tumor samples. Although there are differences between our results and those obtained with breast cancer cells, site specificity and tumor specificity for the role of TGFBR1*6A may be the reason for the difference since TGF-beta signaling differs within tissues of the body.

## Conclusions

This case-control study shows a significant statistical association between TGFBR1*6A variant and osteosarcoma in a Chinese population. The TGFBR1*6A variant is also significantly associated with the distant metastasis of osteosarcoma in the Chinese population studied.

## Competing interests

The authors declare that they have no competing interests.

## Authors' contributions

YSH and YP originally conceptualized the analysis, prepared the samples, and wrote the manuscript. WHL contributed to PCR and gene sequencing studies. YZ help with data analysis. BAM coordinated the study and participated in its design. All authors read and approved the final manuscript.

## Pre-publication history

The pre-publication history for this paper can be accessed here:

http://www.biomedcentral.com/1471-2407/10/169/prepub
